# Airway-Clearance Techniques in Children and Adolescents with Chronic Suppurative Lung Disease and Bronchiectasis

**DOI:** 10.3389/fped.2017.00002

**Published:** 2017-01-24

**Authors:** Annemarie L. Lee, Brenda M. Button, Esta-Lee Tannenbaum

**Affiliations:** ^1^Physiotherapy, School of Allied Health, La Trobe University, Bundoora, VIC, Australia; ^2^Institute for Breathing and Sleep, Austin Health, Heidelberg, VIC, Australia; ^3^Physiotherapy, Alfred Health, Melbourne, VIC, Australia; ^4^Department of Medicine, Monash University, Melbourne, VIC, Australia; ^5^Department of Physiotherapy, Royal Children’s Hospital, Parkville, VIC, Australia

**Keywords:** bronchiectasis, airway-clearance techniques, positive expiratory pressure, mucus production, breathing techniques

## Abstract

Common symptoms of chronic suppurative lung disease or bronchiectasis in children and adolescents are chronic cough with sputum production, retention of excess secretions in dilated airways, and a history of recurrent infections. Clinical management includes the prescription of airway-clearance techniques (ACTs) to facilitate mucociliary clearance, optimize sputum expectoration, relieve symptoms, and improve well-being. A wide range of ACTs are available for selection, and these strategies may be applied in isolation or in combination. The choice of technique will depend in part on the age of the child, their clinical state, and factors which may influence treatment adherence. While the evidence base for ACTs in children and adolescent with these conditions is not robust, the current available evidence in addition to clinical expertise provides guidance for technique prescription and clinical effect. An overview of the most commonly applied ACTs, including their physiological rationale and discussion of factors influencing prescription in children and adolescents is outlined in this review.

## Introduction

Chronic suppurative lung disease (CSLD) is a clinical syndrome in children of respiratory symptoms and signs. Typical symptoms and signs include chronic, productive cough with purulent sputum greater than three episodes per year for longer than 4 weeks. This may be accompanied by exertional dyspnea and recurrent infections (1). In bronchiectasis, unrelated to cystic fibrosis (CF), this clinical presentation is accompanied by radiographic features on high-resolution computed tomography. Common causes of CSLD and bronchiectasis include primary ciliary dyskinesia, humoral immunodeficiency, and chronic aspiration (1, 2). With both conditions characterized by chronic cough and mucus production, airway-clearance techniques (ACTs) are advocated to facilitate the removal of retained secretions (1, 2).

Airway-clearance techniques are widely prescribed in bronchiectasis (3–6), generally for the long term. While often introduced at the time of diagnosis, airway-clearance strategies may adapt and change, according to the presence and frequency of acute exacerbations. No single type of technique is superior to another (7) and physiotherapy prescription of ACTs in children with bronchiectasis is variable (5). While technique selection and prescription is individualized, the chosen ACTs should impose minimal risk of adverse events or aggravation of other symptoms or comorbidities. There are multiple ACTs available, ranging from traditional techniques and those which promote greater independence.

For children and adolescents with CSLD or bronchiectasis, the principles of ACTs follow what has been described for the adult population and for children with CF. This is partially due to the challenges of ACT research, with the need for long-term studies with large patient numbers. Given the paucity of research in the bronchiectasis pediatric and adolescent population, clinical practice has evolved from existing evidence and clinical expertise. However, specific factors which may influence a technique’s effectiveness should be considered when prescribing ACTs for this age group.

Parents of children with bronchiectasis report higher levels of depression (8, 9). Adolescents with bronchiectasis have a higher rate of depression (10). The psychological state of parent and child with CSLD or bronchiectasis may influence adherence to ACT (11) and non-adherence is the primary cause of treatment failure in chronic pediatric lung conditions (11). In addition to child or parental depression, other contributing factors are complexity and demands of a specific treatment routine, lack of support, the need to preserve both family relationships, and a child’s sense of normality (12). Together with an individual’s clinical state, a physiotherapist or respiratory therapist needs to consider these factors when working with a child or adolescent and their family.

This review will provide suggestions for age-appropriate ACTs for children and adolescents with bronchiectasis or CSLD and will outline the most common clinical techniques, the available evidence, and practical considerations for prescription.

## Age Considerations

The choice of ACT should be tailored toward the child’s age. Their level of cooperation, maturity, and psychological adjustment to their condition is all important factors, as are the interactive skills of the physiotherapist and parents (13). Table 1 outlines a guide for age-appropriate ACTs in children and adolescents with bronchiectasis or CSLD. As a child matures, the type of ACT prescribed is likely to change, with greater autonomy in undertaking self-administered techniques.

**Table 1:** **A guide to age-appropriate airway-clearance techniques for children and adolescents with bronchiectasis or chronic suppurative lung disease**.

Technique	Age range	Advantages	Disadvantages
Modified GAD or GAD	All age ranges	Suitable for infants and small children who are not yet old enough to cooperate with more active techniques	Discomfort, time consuming, symptoms of gastro-esophageal reflux or breathlessness, specific contraindications or precautions
		Option for those unable to use or too fatigued to use independent techniques	

Percussion and vibrations	All age ranges	Suitable for infants and small children who are not yet old enough to cooperate with more active techniques	Passive, require assistant, discomfort, inconvenient, socially limiting
		Option for those unable to use or too fatigued to use independent techniques	

Assisted autogenic drainage	Infants	Minimal equipment required	Requires assistance, difficult technique to master and for carers to learn

Bouncing on a fitball	Infant to toddler	Enjoyable for child	Equipment required

Blowing games	Toddler to child	Enjoyable for child	

Huffing	Toddler to adolescent	Enjoyable for child	

ACBT (includes huffing)	Toddler to adolescent	Independent, flexible, requires no equipment, can be combined with other techniques	

Bottle PEP	Toddler to adolescent	Independent technique, enjoyable for child, can be a bridge to other forms of PEP therapy, minimal cost	Need to follow instructions to avoid swallowing water

Autogenic drainage	Toddler to adolescent	Independent technique, nil equipment required	Effect and feedback required to master the technique including sensitivity to auditory and vibratory cues of secretions

PEP mask	Toddler to adolescent	Independent technique, can be combined with other ACTs, beneficial for those with unstable or compliant airways	Infant PEP requires assistance. Requires individual awareness of breath size
			For younger children who are afraid of a mask, this may not be the technique of choice
			Cost

Mouthpiece PEP	Toddler to adolescent	Independent technique, can be combined with other ACTs, beneficial for those with unstable or compliant airways	No clear evidence on use of Mouthpiece PEP—either with or without nose clip Cost
		Can be used in conjunction with hypertonic saline nebulizer (see below)	
		Easy for younger children to use	

PariPEP™ with nebulizer	Toddler to adolescent	Independent technique, can be combined with other ACTs, beneficial for those with unstable or compliant airways	Cost

Flutter^®^	Child to adolescent	Independent technique	Effective use dependent on angle, therefore, may be more suited to an older child (8 years). Cost

Acapella^®^	Toddler to adolescent	Independent technique, can be combined with other ACTs, beneficial for those with unstable or compliant airways	Cost
		Not position dependent	

Aerobika™	Toddler to adolescent	Independent technique, can be used in conjunction with nebulizer	Cost

Physical exercise	Toddler to adolescent	Enjoyable for child	

HFCWO	Toddler to adolescent	Independent technique	Heavy device, not easily portable
			Cost

*GAD, gravity-assisted drainage; ACBT, active cycle of breathing technique; PEP, positive expiratory pressure; ACT, airway-clearance technique; HFCWO, high-frequency chest wall oscillations*.

## Types of Techniques

### Gravity-Assisted Drainage (GAD) and Manual Techniques

Gravity-assisted drainage involves placing the patient in specific (including semi-recumbent) positions which enables gravity to drain excess secretions from bronchopulmonary segments (14, 15). Infants and toddlers may be positioned on a caregivers’ lap, while older children and adolescents use a couch/bed. While traditional positioning involves a head-down tilt in selected positions, gastro-esophageal reflux (GOR) in infants and children with CF (16–19) has led to adopting a modified GAD approach (ModGAD), which eliminates a head-down tip for those suspected of or demonstrating GOR (5, 18). Despite no evidence that GAD aggravates GOR in children or adolescents with bronchiectasis, the presence of this comorbidity (20, 21) implies the potential need for applying ModGAD as required. In addition, ModGAD has demonstrated equivalent effectiveness as traditional GAD and is preferred by patients with bronchiectasis (22).

This technique is often combined with manual techniques, such as chest percussion or vibrations, with the technique application adjusted for the child’s age (23, 24). The application of force to the chest wall creates changes in the intrapleural pressure, which are transmitted through the thoracic cage and assist in dislodging secretions. Chest wall vibrations apply fine oscillatory movements with chest wall compression, initiated at the end of inspiration and applied during expiration.

Despite the lack of focused research of these techniques in children and adolescents with bronchiectasis, GAD or more commonly ModGAD improves secretion clearance and, combined with manual techniques, is equally effective as other ACTs (25–27). Some studies of individuals with bronchiectasis demonstrate greater sputum expectoration with GAD and manual techniques compared to oscillating positive expiratory pressure (PEP) therapy or the active cycle of breathing technique (ACBT) (22, 28). The application of ModGAD with percussion and/or vibrations is most commonly applied in infants and young children or reserved for older individuals who cannot manage their own treatment.

### Active Cycle of Breathing Technique

The ACBT consists of thoracic expansion exercises and the forced expiratory technique (FET) (29, 30). The technique is applied in an upright sitting, ModGAD, or other recumbent positions. Thoracic expansion exercises are deep breathing exercises, with an emphasis on slow, controlled inspiration. Inspiration is believed to facilitate collateral channel ventilation to reach behind secretions. Breathing control is tidal volume breathing to relieve breathlessness, which may be generated during more active components of ACBT (31). The FET is a key component of ACBT. It is a combination of one to two forced expirations (huffs) and breathing control. The huff comprises of controlled expiration through an open glottis to near low volumes. During a forced expiration, the equal pressure point moves peripherally into the smaller airways. Secretions are mobilized by commencing huffing at low, mid, and high lung volumes. FET maneuvers are the most effective part of chest physiotherapy (32), with this technique frequently applied with other ACTs (33).

**Figure 1:**
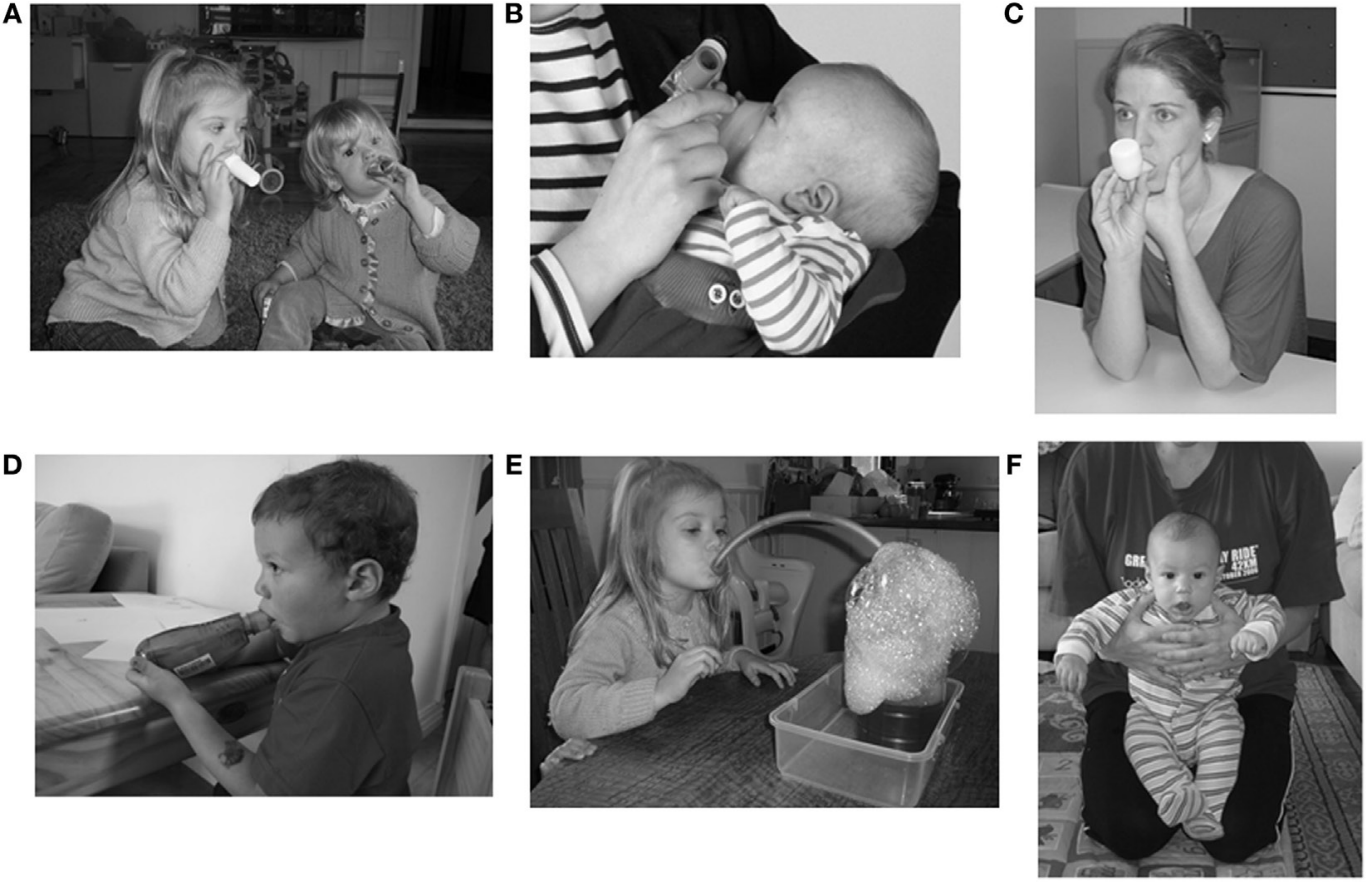
**Examples of airway-clearance techniques: (A) huffing, (B) infant positive expiratory pressure (PEP) mask, (C) oscillating PEP (OscPEP) with Flutter^®^, (D) OscPEP with Acapella^®^, (E) bottle PEP, (F) assisted autogenic drainage**. Permission to publish images has been granted by individuals or on behalf of the individuals.

To encourage glottis opening, a peak-flow mouthpiece or similar piece of tubing may be used, providing audible feedback to children learning the technique (Figure 1A). Commencing huffing games in toddlers is a useful initial strategy to encourage the correct technique.

Only studies of adults with bronchiectasis undertaking ACBT (in recumbent positions) have been conducted, and when compared to other techniques, similar improvements in quality of life and lung function have been consistently noted (28, 34, 35).

### Positive Expiratory Pressure (Therapy)

Positive expiratory pressure therapy uses a one-way valve that allows unrestricted inspiration and a resistance to expiration. The theoretical rationale for PEP is that in the presence of small airway obstruction, PEP therapy promotes airflow past the obstruction or through the collateral channels. This allows an increased volume of air to accumulate behind secretions while the pressure gradient across the sputum plug forces secretions toward the larger airways (36, 37). During expiration, the positive pressure is thought to increase functional residual capacity, preventing premature airway collapse (38). Breathing cycles are coupled with FET as required.

A commonly applied PEP therapy system consists of a close-fitting mask and a one-way valve to which expiratory resistors are attached. Alternatively, a mouthpiece with holes of varying diameters (PariPEP™) to apply expiratory resistance or a TheraPEP^®^ is used. A manometer determines the correct pressure generated during initial therapy instruction. For low pressure PEP therapy, the resistor giving a pressure level of 10–25 cmH_2_O during the middle of expiration is an ideal selection (39, 40). While PEP therapy may be used in young children and adolescents of all ages (from infancy to older age), there is a slight variation with infant PEP, with an appropriately sized face mask held over the infant’s nose and mouth (Figure 1B). Generation of specific pressures is not a goal of infant PEP therapy due to poorly developed collateral ventilation. It is often combined with a physical activity, such as sitting and bouncing on a fit ball, with the additional activity modulating lung volumes. For younger children afraid of a mask, PEP mask is not the technique of choice.

Most studies of PEP therapy in children and adolescents have been conducted in CF. Short-term PEP therapy is associated with similar sputum expectoration, lung function, and well-being as other ACTs, although PEP is a preferred technique (41). One long-term study of PEP for 12 months in children with CF concluded that PEP was superior to GAD with manual techniques (42). In infants with CF, twice daily infant PEP was safe and improved gas exchange compared to ModGAD over a 12-month period (43).

### Oscillating PEP (OscPEP) Therapy

Oscillating PEP therapy offers the combination of PEP with high-frequency oscillations which elicit shear forces within the airways during exhalation to facilitate secretion clearance. The oscillations are believed to induce vibrations within the airway wall to displace secretions and the repeated accelerations of expiratory airflow favor movement of secretions from the peripheral to the central airways (44). There are several devices which provide OscPEP therapy, with the Flutter^®^ and Acapella^®^ among the most common. Other devices which provide similar effects are the Aerobika™, the Quake, and the RC-Cornet^®^.

The Flutter^®^ is a small pipe-shaped hand-held device with a mouthpiece, a perforated cover which encases a stainless steel ball resting in a circular cone. Inhalation occurs through the nose or around the mouthpiece. During expiration, at a slightly faster rate than normal, the high-density ball rolls up and down the cone, creating interruptions in expiratory flow and generating a PEP within the range of 18–35 cmH_2_O (45). An oscillatory vibration of the air is generated which shear secretions from the airways and reduces the viscoelasticity of the secretions (44). Cycles of inspiration and expiration are repeated, followed by FET and coughing as required. The frequency of oscillations is determined by the angle at which the device is held (46). It can be difficult for children to maintain the angle position for an extended length of time and consistently achieve maximal effect. Optimal technique may be more evident with older children (8 years and up) (Figure 1C).

### Acapella^®^

The Acapella^®^ uses a counterweighted plug and magnet to create airflow oscillations (47). The frequency/resistance dial allows adjustments to the expiratory pressure and the oscillation frequency, with the device used with either a mouthpiece or mask. Inspiration can occur through the nose or mouth, with an inspiratory pause and a more active exhalation, followed by FET. The Acapella^®^ is flexible in the positions in which it can be used (upright and recumbent). In children, the resistance level is set low initially (number 1 or 2) and then increased slowly as necessary. When instructing a toddler or child, 5 or 10 breaths may be an initial attempt. Once the child masters the blowing technique, a more formal number of breaths and frequency is provided (Figure 1D).

### Other OscPEP Devices

The RC-Cornet^®^ consists of a mouthpiece, curved tube, a valve hose, and a sound damper, with expiration through the tube creating an increasing pressure within the hose until it opens, allowing air to flow through the device and creating a PEP and vibrations within the airways. Similar to an Acapella, the Aerobika™ includes a mouthpiece and one-way valve. Nebulizer therapy can also be incorporated into the circuit as well to deliver mucolytic therapy.

The majority of research of OscPEP in bronchiectasis has focused on the Flutter^®^, Acapella^®^, and RC-Cornet^®^ in adults. When applied in the short term (single treatment session) or long term (4 weeks or 3 months), OscPEP improved sputum expectoration and HRQOL compared to no treatment (25, 48). However, when compared to other ACTs, the effects upon sputum expectoration, spirometry, gas exchange, and symptoms were equivalent (28, 34, 35, 49), although its independence is preferred by patients (28). Considering the degree of monitoring required of the child’s technique to minimize airway closure may be important factors in selecting OscPEP therapy for some children.

### Bottle PEP

Bottle or Bubble PEP is an alternative method to administer low pressure OscPEP therapy, particularly for children less than 4 years, who no longer tolerate infant PEP but are not yet able to progress to other forms of ACT. The resistance in this set up is a water column, with the expiratory pressure remaining constant once the tubing diameter is >5 mm (50). It consists of smooth bore rubber tubing and a plastic bottle (1–2 L) that is approximately ½ filled with water. The child inhales through his/her nose or around the tube in his/her mouth and expires through the tube into the column of water. Blowing through the tubing creates bubbles in the bottle. The height of the water (approximately 10 cm above the bottom of the tube) provides the PEP while the bubbling produces oscillations in the airways. For children, the addition of liquid detergent and a small quantity of food coloring adds to the novelty of the treatment (Figure 1E). For children with bronchiectasis and a comorbidity (i.e., cerebral palsy), a facemask Bottle PEP can be set up with a one-way valve. Initial use with toddlers/children may allow blowing as many times as they wish to see that they are creating bubbles and maintain engagement. Once the blowing is mastered, a more formal number of breaths and sets are instructed. Despite use in clinical practice (6, 51–54), the evidence for Bottle PEP is scarce and consideration regarding approval as a medical device may be necessary (55).

### Autogenic Drainage (AD)

Autogenic drainage is a technique which maximizes airflow to promote ventilation and secretion clearance. It employs the principles of breathing at different lung volumes to loosen and mobilize secretions (56). The aim is to achieve the highest possible expiratory airflow while avoiding dynamic airway collapse (57). The speed of the expiratory flow reduces mucus adhesion, shears secretions from bronchial walls, and transports them from the peripheral to proximal airways (58). AD consists of three phases and the duration of each phase will depend on the efficacy of airflow to mobilize secretions.

In those with bronchiectasis, a single session of AD cleared more secretions compared to no treatment (59). Long-term studies in children with CF comparing AD to OscPEP, PEP mask, or ACBT have demonstrated clinical equivalence between ACTs (33). In adolescents, although clinical benefit was similar, a preference for AD over GAD and manual techniques was evident over the 2-year study (60). This highlights the importance of patient preference and its impact on adherence (61). While AD is flexible, it is a complex and technically difficult treatment strategy. Requiring patience and cooperation, it takes time for child or adolescent to learn and utilize feedback to adequately perform the technique. For this reason, it is not suitable for young children.

An alternative adaptation is assisted AD, which may be applied for infants and young children. Assisted AD is achieved by applying gentle manual pressure to the child’s chest wall by the physiotherapist/parent/caregivers’ hands to increase expiratory flow and achieve different lung volume breathing (55). The infant or child is supported on a physiotherapist/caregiver’s lap, who is often sitting on a fit ball. Assisted AD is performed in a gentle and progressive way, using the patient’s breathing pattern; the option of bouncing on a fit ball encourages relaxation of the child and enhances expiratory air velocity (Figure 1F). As an infant or child needs to remain still for a period of time for the technique to be effective, it can be difficult to use for some children.

### High-Frequency Chest Wall Oscillation (HFCWO) and Intrapulmonary Percussion

High-frequency chest wall oscillation applies external chest wall oscillations *via* an inflatable vest worn around the torso (62). Increasing the air-liquid shear forces during expiration is believed to result in secretion mobilization. Although only applied in adults with bronchiectasis, the HFCWO improved pulmonary function and quality of life and reduced dyspnea compared to GAD with breathing techniques (63). Although unstudied in pediatric populations with bronchiectasis, it is an option if the cost is not prohibitive and has been applied in children with neurological conditions and bronchiectasis, with a reduced incidence of pneumonia over 12 months (64). Other options include intrapulmonary percussive ventilation, which provides high-frequency oscillatory ventilation to produce endotracheal percussion. Models of acoustic percussion (The Frequencer™ and Vibralung^®^) induce vibrations in the chest wall and are an alternative option to therapist-delivered manual techniques. However, there is minimal evidence of their clinical effects in the bronchiectasis or CSLD pediatric populations.

### Exercise

Physical exercise, particularly endurance activity, is highly recommended for all ages in the management of bronchiectasis and CSLD (65), with endurance and muscle and bone strengthening activities included. Activity selection is age-dependent and incorporated into school sporting or external activities where possible. Ideally, toddlers and young children have fun, physical activities interspersed with other ACTs (66). For older children, cycles of exercise of a moderate intensity are mixed with ACBT, AD, PEP therapy, or FET. Not only does it promote general health and well-being but exercise is a more effective bronchodilator compared to beta-agonists in primary ciliary dyskinesia (65).

### Adjuncts to ACTs

Other options which may facilitate ACTs include mucoactive agents, delivered by nebulized inhalation therapy. Hypertonic saline is classed as an expectorant and may be inhaled before or during an ACT (2). It increases airway surface hydration to reduce secretion viscosity (67). Although beneficial in adults with bronchiectasis, improving lung function (68), the superiority of hypertonic saline over isotonic saline is unclear (69). While not recommended for routine use (1), a therapeutic trial in children experiencing frequent acute exacerbations can be considered.

## Conclusion

A key component of managing CSLD or bronchiectasis in children and adolescents involves ACTs and exercise and although the evidence base is lacking, clinical practice reflects their regular prescription and use. Technique choice will vary according to age and specific factors influencing patient adherence. Working closely with the child or adolescent and their family in a therapeutic alliance and providing ongoing education, engagement, and encouragement to assist with adherence to therapy is critical in maximizing the effectiveness of airway-clearance therapy in these populations.

## Author Contributions

AL drafted the manuscript, BB and E-LT provided critical input to the manuscript, and all the authors approved the final version.

## Conflict of Interest Statement

The authors declare that the research was conducted in the absence of any commercial or financial relationships that could be construed as a potential conflict of interest.
